# Using ^13^C in cattle hair to trace back the maize level in the feeding regime—A field test

**DOI:** 10.1371/journal.pone.0188926

**Published:** 2017-11-28

**Authors:** Verena Hammes, Olaf Nüsse, Johannes Isselstein, Manfred Kayser

**Affiliations:** 1 Georg-August-University of Goettingen, Department of Crop Sciences, Grassland Science, Location Vechta, Vechta, Germany; 2 Georg-August-University of Goettingen, Department of Crop Sciences, Grassland Science, Goettingen, Germany; 3 Georg-August-University of Goettingen, Centre of Biodiversity and Sustainable Land Use, Goettingen, Germany; University of Oregon, UNITED STATES

## Abstract

Sections from cattle hair serve as an isotopic archive—they contain information on the cattle diet from different time periods. We tested the reliability of ^13^C signatures (δ^13^C) in cattle tail switch hair to retrospectively trace back the annual dietary proportion of maize of different production systems without having to sample and analyze the feed. Furthermore, we investigated if differences in dietary proportion of maize during summer and winter feeding can be detected in a single tail switch hair by sampling hair only once a year. We sampled hair and obtained information on management and annual composition of diets on 23 cattle farms in northern Germany. Farms differed in dietary proportions of maize, grass and concentrates as well as in grazing regime (year-round grazing, summer grazing, no grazing). We found that the annual mean δ^13^C values (δ^13^CY) of two hair sections that contain the isotopic information of summer and winter feeding is a robust indicator for the annual proportion of maize in cattle diet on a farm. The grazing regimes could clearly be distinguished by analyzing seasonal mean δ^13^C values (δ^13^CS). We could also demonstrate short term changes in the diet changes by means of δ^13^CS. We conclude that the method can be used in different cattle production systems to check on dietary proportions of maize for a period of one year before sampling of hair.

## Introduction

Currently, in dairy cows there seems to be an on-going trend to increasing milk yields which require higher energy contents in the diet that are often provided by silage maize and concentrates. Maize is comparably easy to cultivate at reasonable costs and the area that is cultivated with maize for dairy production in Germany has increased during the last half century [1, 2]. Consequently, maize is often cultivated in intensive cattle production systems with N surpluses resulting in a potential risk for larger N emissions compared to grassland-based forage production [3]. The public interest in food production methods and the origin of food has been increasing over the last years [4–6]. In livestock production, pasture-based systems are regarded as more friendly for the environment, as promoting animal-welfare, and are thought to yield healthier products compared to non-grazing systems [7–10]. To verify the feeding regimes of farms, simple and robust indicators would be needed [11, 12]. However, most cattle farms, especially dairy, feed a mixture of grass silage, maize and concentrates in the daily diet of their animal and in practice, it is very difficult to obtain detailed and reliable information on the feeding regimes of farms [13, 14].

Isotopic analysis of different animal tissues and products has been successfully used to evaluate food quality, to retrace animal feeding and to assess production systems in general in recent years [15–20]. It has been confirmed by isotope ratio mass spectrometry (IRMS) that the isotopic signature of cattle diet is transferred to animal tissue [21–24]. In this respect, hair proved to be especially useful as it grows more or less continuously [25–28] and can preserve isotopic information for many years [29]. Thus, hair can serve as an isotopic archive recording dietary changes over time [30]. Furthermore, sampling of hair is quick and easy and can be done without much disturbance to the animal [31].

The carbon isotopic signatures (δ^13^C) of C_4_ plants, such as maize, differ strongly from that of C_3_ plants (grasses, legumes, concentrates like soy or grain) [15] because of different photosynthetic pathways. Therefore, differences in the dietary proportion of C_3_ and C_4_ plants are related to the δ^13^C in cattle tissue [32–34]. The strong correlation of δ^13^C in cattle tissue and the dietary proportion of maize has been shown in a number of studies [6, 15, 21, 35, 36]. In this study, we sampled cattle hair once a year in various cattle production systems in northern Germany and analyzed δ^13^C of two hair sections that contained isotopic information of the summer and winter periods of the previous year. Firstly, we tested the reliability of the use of annual mean δ^13^C values of both hair sections as a tool for tracing back the annual mean proportion of maize fed. Secondly, we tested the reliability of the use of seasonal mean δ^13^C values, that are the separate hair sections representing summer and winter, as a tool for confirming the grazing regime (year-round grazing, summer grazing, no grazing) and for tracing back changes in the dietary proportions of maize between summer and winter feeding periods.

The farms in our survey were chosen along a location gradient (longitude, temperature, precipitation) and a production intensity gradient. During farm visits we asked farmers for information on the mean dietary proportions of maize, grass (silage, fodder from pasture, hay) and concentrates in their rations for cows during the year before sampling the tail hair and on the grazing regime (year round grazing, summer grazing, no grazing).

## Material and methods

### Sampling design

For isotopic analysis of cattle hair and the related feeding regimes, we chose 23 cattle farms, mainly dairy but also suckler cows on the basis of two gradients (Table 1): 1) a location gradient (longitude, temperature, precipitation) and 2) a production intensity gradient characterized by the annual milk yield per cow per year and the stocking rate. Suckler cows are kept for the production of beef, rather than milk. Their calves stay with the herd and are fed by the cow until the calves are ready to be sold either for fattening or to a meat factory for beef. For estimating the milk yield of suckler farms we used standard values from the literature [37]. Sampling of cattle hair was carried out in 2014 and obtained the isotopic information referred to cattle feeding in 2013.

**Table 1:** Details of the production systems of cattle farms in the survey on a gradient from west to east.

					Annual dietary proportions (% DM)			
Farm	Farm type	Lon	Milk yield (kg/cow/yr)*	Grazing Regime	Maize	Grass (pasture, silage, hay and haylage)	Concentrates	Stocking rate (LU/ha)*
A	Dairy	8.69	7300	Summer	0	71	29	0.6
B	Dairy	8.37	11212	No grazing	39	36	25	0.9
C	Suckler cow	8.62	2500	Year-round	0	96	4	1.6
D	Dairy	8.33	9500	Summer	35	35	30	1.4
E	Suckler cow	8.64	2500	Year-round	0	100	0	1.0
F	Suckler cow	10.67	2500	Year-round	0	100	0	0.8
G	Suckler cow	10.30	2500	Year-round	0	98	2	0.6
H	Dairy	10.48	9600	No grazing	53	27	20	1.9
I	Dairy	10.74	9000	Summer	36	36	28	0.3
J	Dairy	10.54	8800	No grazing	35	45	20	2.5
K	Dairy	10.67	8845	No grazing	39	17	44	2.0
L	Dairy	12.64	7300	Summer	54	29	17	0.7
M	Suckler cow	12.85	2500	Year-round	0	100	0	0.3
N	Dairy	12.09	8500	No grazing	20	59	21	1.1
O	Dairy	12.74	7000	No grazing	36	27	37	0.5
P	Dairy	12.14	9760	No grazing	39	26	35	1.8
Q	Suckler cow	12.69	2500	Year-round	0	100	0	0.3
R	Suckler cow	13.87	2500	Year-round	0	98	2	0.4
S	Dairy	14.64	11266	No grazing	45	20	35	0.5
T	Dairy	14.20	9870	No grazing	50	25	25	0.5
U	Dairy	14.10	9550	No grazing	55	19	26	0.6
V	Dairy	14.09	8870	No grazing	64	0	36	0.2
W	Dairy	13.90	6200	Summer	31	53	16	1.2

* We used standard values for estimating the milk yield of suckler cow farms (KTBL, 2009) Stocking rate: LU/ha of agricultural land. LU = livestock unit, 500 kg of body weight; Lon = Longitude (°).

To describe the feeding regime on the farms, we developed a questionnaire to be answered in face-to-face interviews with the farm managers. Farmers gave information on annual dietary proportions and grazing regimes for the year 2013 (Tables 1 and 2). Maize was always the only C_4_ plant in cattle diets. The concentrates that were fed on the farms did not contain any C_4_ components, it consisted of soybean meal, sugar beet molasses, and wheat and barley seed.

**Table 2:** Recorded variables, source and range.

Variable	Unit	Source	Range
Grazing regime		Questionnaire	1) Year-round grazing, 2) Summer grazing, 3) No grazing
Farm size (Agr. land*)	ha	Questionnaire	20–3800
Arable Land	ha	Questionnaire	0–3500
Grassland area	ha	Questionnaire	8.3–450
Precipitation:			
Annual precipitation	mm	Weather station (DWD)	525–693
Precipitation during vegetation period, April-October	mm	Weather station (DWD)	351–482
Temperature:			
Annual mean temperature	°C	Weather station (DWD)	8.9–9.3
Mean temperature during vegetation period, April-October	°C	Weather station (DWD)	13.9–14.9
Length of vegetation period	d	Detection station (DWD)	171–179

*Agr. land = Agricultural land

DWD = Deutscher Wetter Dienst, German Weather Service.

The farms could be assigned to three grazing regimes: year-round grazing, summer grazing (May–October), and no grazing. In the year-round grazing regime, cattle did not receive any maize; in the summer-grazing regime, cattle were kept in the stable during the night and for milking and received additional feeds like maize silage and concentrates during this time. In the no grazing regime the cattle were kept in stables throughout the year and were fed a diet that always contained maize. The breeds included Holstein, Deutsche Schwarzbunte, Deutsches Fleckvieh and Welsh Black. We assumed that cattle breed has no influence on the isotopic signatures in the cattle hair [31]. Growth rates of cattle tail switch hair can differ to a small extent among cattle breeds and among individuals [31]. However, assuming a medium growth rate of 0.8 mm/day and using hair sections of 1 cm in length in the isotopic analysis is a good compromise between precision/resolution and effort [28, 31, 38]. Differences in δ^13^C of grass silage, hay and fresh grass from temperate humid pasture are negligible [21].

Data on temperature and precipitation were obtained from the Climate Data Center (CDC) of the Deutsche Wetterdienst (DWD) (Table 2).

### Study area and farms

Our study area was located between 51°50’ to 53°05’ North and 8°19’ to 14°38’ East in northern Germany in an area called North German Plain (NGP). The NGP is a part of the geomorphic formation called North European Plain (NEP, elevation 0 to 200 m above sea level) which stretches from the Netherlands to Poland/Lithuania. To the South the NEP is confined by the Central European Highlands while to the west is the North Sea and to the east the Baltic Sea. The climatic conditions range from sub-maritime in the west to sub-continental in the east.

All farms are located on sandy soils between 38 and 76 m above sea level. The mean annual precipitation in the year 2013 varied between 525 and 693 mm and mean annual air temperature ranged between 8.9 and 9.3°C (Table 2).

### Hair sampling and preparation

The tail switch hair is the longest hair of cattle and is therefore best suited for detecting changes in diet over long periods in the past [31]. We plucked bundles of approximately 50 cattle tail switch hairs from two full grown, healthy animals of average productivity of each farm from March 10 to March 20, 2014. All sampled animals had been kept on the farm for more than 2 years before sampling. After sampling, the hairs were put in bags and were frozen for storage until further processing. We prepared the hair samples for stable isotope analysis according as follows [21, 31]: To remove contaminants, like traces of faeces, the hairs were soaked and washed by ultra-sonication with deionized water, dried (40°C, 48 h), soaked in a 2:1 mixture of methanol/chloroform (approximately 2 h), rinsed with deionized water, soaked in deionized water for another 30 min, and rinsed again; finally, the hairs were dried again (40°C, 48 h).

### Position-time conversion and hair growth phases

Mammal hair undergoes distinct phases of growth (anagen phase) and rest (telogen phase) [39]. In order to clearly assign a certain segment of the hair to a certain period of growth and the respective diet at that time, we needed to know if the hair was in the growing or resting phase when plucked. In our study, we distinguished between anagen and telogen hair by microscopic examination as described in literature [21, 40] and selected only three anagen hairs of similar length from each animal for further isotopic analysis.

For the interpretation of the isotopic analysis, we assumed a medium hair growth rate of 0.8 mm/day and the isotopic signal of a new diet after a diet switch to take 80 days to be clearly detectable in cattle hair [28, 31, 38, 41–44]. This implies that each part of a hair could be assigned to an approximate period during which it had grown [22, 24].

**Fig 1:**
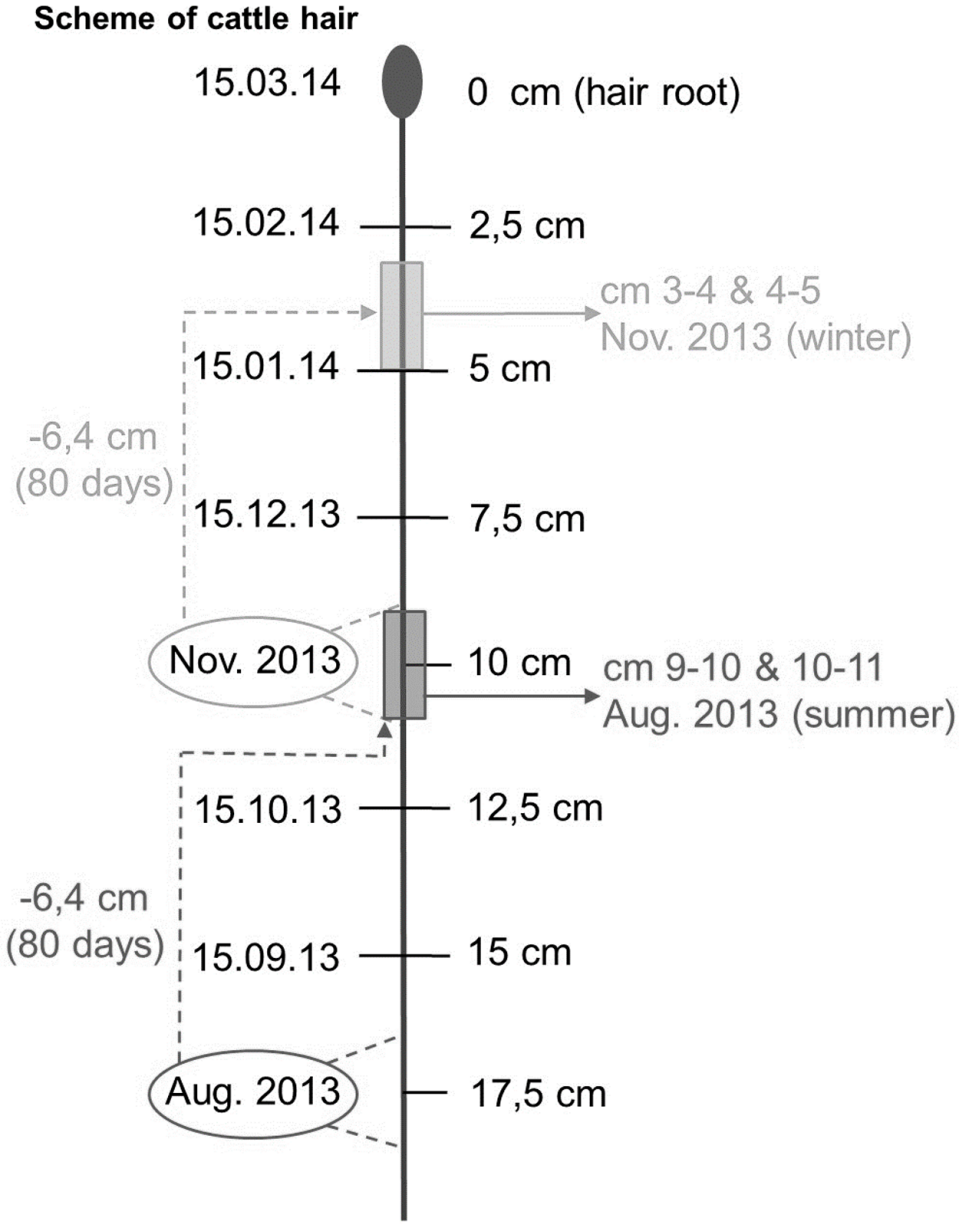
Scheme of cattle hair plucked on March 15, 2014 showing hair sections that were analyzed for the winter (light grey) and summer period (dark grey).

From a bunch of approximately 50 tail hairs plucked from each cow three hairs were randomly chosen for isotopic analysis. We selected four hair segments of 1 cm each, two for each season (Fig 1) from each of the three cattle hairs. We then arrived at 12 data points per farm per season. For the summer period (August 2013) we chose the 9–11 cm section and for the winter period 2013 (November 2013) the 3–5 cm section. Each segment of 1 cm was cut with a stencil into even smaller pieces and put into a tin cup (4x6 mm) for isotopic analysis.

The response of cattle hair to a dietary signal can be described by an exponential decay function [31]. The diet of the last days before hair growth contributes the largest share to the signal of the newly grown hair section. Our selected section for the summer period therefore contained around 50% isotopic information of the summer—the grazing period (October, September) and another 50% of the winter period—the stable period (November).

Cattle tail switch hair, especially of dairy cows, is regularly cut for hygienic reasons and hairs are often only about 10 cm long. We plucked hair in mid-March 2014 and chose the oldest hair section in order to get an isotopic signal from the previous summer that was as clear as possible. In fact, the sampling of the hair section that represented the summer period (9–11 cm from the hair base) has been a compromise between practical application and precision—sections from tail hair of at least 12 cm would have given an even clearer signal. In our study, the average length of suckler cow tail hair was 32.4 cm and that of dairy cow tail hair was 13.5 cm; the shortest hair was 11.3 cm.

### Isotopic analysis

The isotopic analysis was carried out with an isotope ratio mass spectrometer Delta Plus IRMS linked with a Conflo III-Interface (Finnigan MAT, Bremen, Germany) to an elemental analyzer NA1110 (Carlo Erba Instruments, Milano, Italy). The isotope data are presented in parts per thousand (‰) as δ^13^C (‰), with δ^13^C = [(R_sample_ / R_standard_)-1] x 10^3^ and R the ^13^C/^12^C ratio in the sample or standard (V-PDB, “Vienna Pee Dee Belemnite“). Each sample was measured against a secondary laboratory standard (Acetanilid) which had previously been calibrated against international standards (IAEA NBS18 and IAEA 600); this resulted in a reference value of -29.96 ‰. The precision (SD) for the internal lab standards was 0.28%.

### Statistics

In the first step, we looked for possible correlations amongst the parameters to get information on the structure of our dataset. In the second step, we used a linear model based on annual mean δ^13^C values of each farm, which are referred to as δ^13^CY (Y = year; n = 23 δ^13^CY values), to test the reliability of the general relationship of annual δ^13^C in cattle hair and the annual mean proportion of maize. In the third step, we used seasonal data in a two-way analysis of variance (ANOVA) to asses for effects of grazing regime and seasons (summer and winter) on δ^13^C values in cattle hair. This was followed by a Tukey post hoc test to compare means of grazing regimes and seasons. Seasonal mean δ^13^C values are referred to as δ^13^CS (S = season; n = 46 δ^13^CS values. We calculated the means for the summer and winter period by averaging the 12 data points of each season from each of the 23 farms (2 cows, 3 hairs, 2 segments per season) resulting in 23 values for the summer and 23 values for the winter period per farm. We used box plots to indicate variability within groups and as a basis for discussing outliers. For all statistical analyses we used the R software (version 3.2.1, packages ‘nnet’ and ‘car’).

## Results

### Correlations between δ^13^CY in cattle hair and production systems’ characteristics

The δ^13^CY values in cattle tail switch hair of each farm were highly correlated with the annual dietary proportion of maize (Table 3). Also the dietary proportions of grass and concentrates were correlated with δ^13^CY in cattle hair because the dietary proportions of maize, grass and concentrates were highly intercorrelated (Table 3). The milk yield per cow was correlated with δ^13^CY; this relation being due to the strong positive effect of maize on the milk performance of the cattle. We did not find any correlation between δ^13^CY values in cattle hair and the longitude, temperature or precipitation (Table 3).

**Table 3:** Correlation coefficients between δ^13^CY values in cattle hair of each farm and selected parameters of cattle production systems and location of the farms.

	δ^13^CY	Maize in diet (%)	Grass in diet (%)	Concentrates in diet (%)	Milk yield (kg/cow/yr)	Longitude (°)	vegP (mm)	vegT(°C)
δ^13^CY	1							
Maize in diet (%)	0.95	1						
Grass in diet (%)	0.94	-0.97	1					
Concentrates in diet (%)	0.84	0.83	-0.94	1				
Milk yield (kg/cow/yr)	0.84	0.85	-0.87	0.83	1			
Longitude (°)	0.33	0.37	−0.33	0.25	0.11	1		
vegP (mm)	0.00	−0.05	0.02	0.02	−0.09	−0.17	1	
vegT (°C)	0.14	0.22	−0.20	0.16	0.04	0.84	−0.36	1

vegP = precipitation during vegetation period (April-October); vegT = Temperature during vegetation period (April-October). Light grey numbers indicate low correlations.

### The relationship between mean δ^13^CY in cattle hair and annual dietary proportion of maize

**Fig 2:**
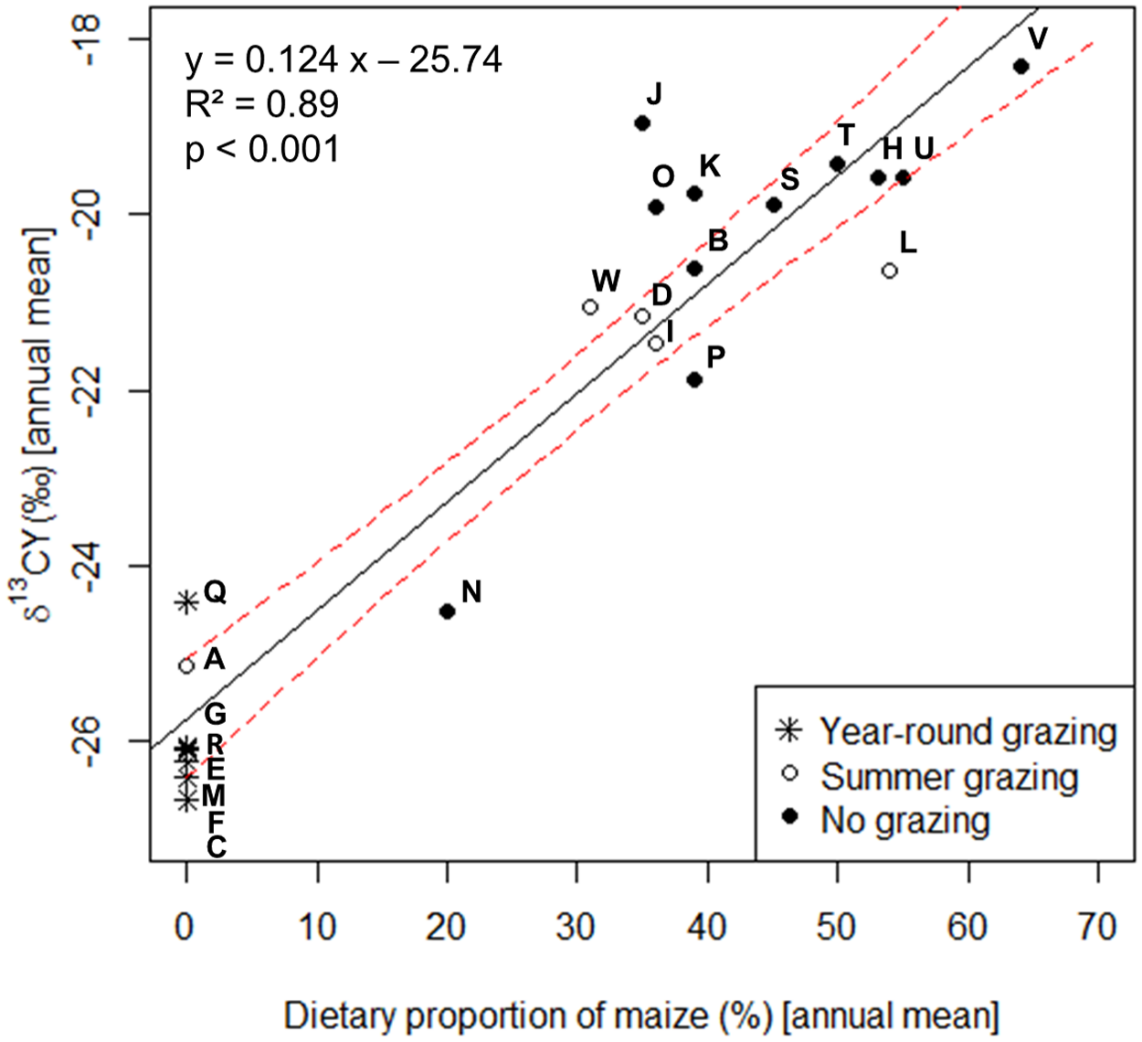
Relationship between δ^13^CY values in cattle hair and annual dietary proportion of maize (% dry matter). Each point refers to a farm. The letters A-W indicate the farms (see Table 1). The solid and dotted lines refer to regression line and 95% confidence interval (CI), respectively (RSE = 0.98).

In the second step, we used a linear model to analyze the relationship between the annual dietary proportion of maize and the δ^13^CY values in cattle hair. We found that 89% (p<0.001) of the variation in δ^13^CY in cattle hair could be explained by the annual dietary proportion of maize (Fig 2). The intercept of our linear model is at -25.74 ‰ δ^13^CY with the confidence intervals (CI 95%) at -26.42 and -25.10; the estimate for the annual dietary proportion of maize (%) amounts to 0.124 ‰ δ^13^CY (CI 95% = 0.10; 0.143); the residual standard error is 0.98. Our model confirms that a δ^13^CY value in cattle hair higher than -25.10 ‰, that is the upper limit of the confidences interval taken as a conservative value, is an indication for maize in the cattle diet of up to one year before hair sampling (Fig 2). A proportion of 20% maize in the annual diet corresponds to an estimated δ^13^CY value of -23.26 ‰ with a lower CI 95% at -24.32 ‰ and the upper at -22.24 ‰; values for δ^13^CY for 60% maize in the diet would amount to -18.30 ‰ (CI 95% = -20.12 ‰; -16.52 ‰).

### Differences in δ^13^CS values in cattle hair among grazing regimes and seasons

The three grazing regimes differed significantly in mean δ^13^CS in cattle hair (p < 0.001): year-round grazing -26.00 ‰; summer grazing: -21.90 ‰; no grazing: -20.22 ‰. We found no significant difference between the seasons and no significant effect of the interaction of grazing regime and season.

**Fig 3:**
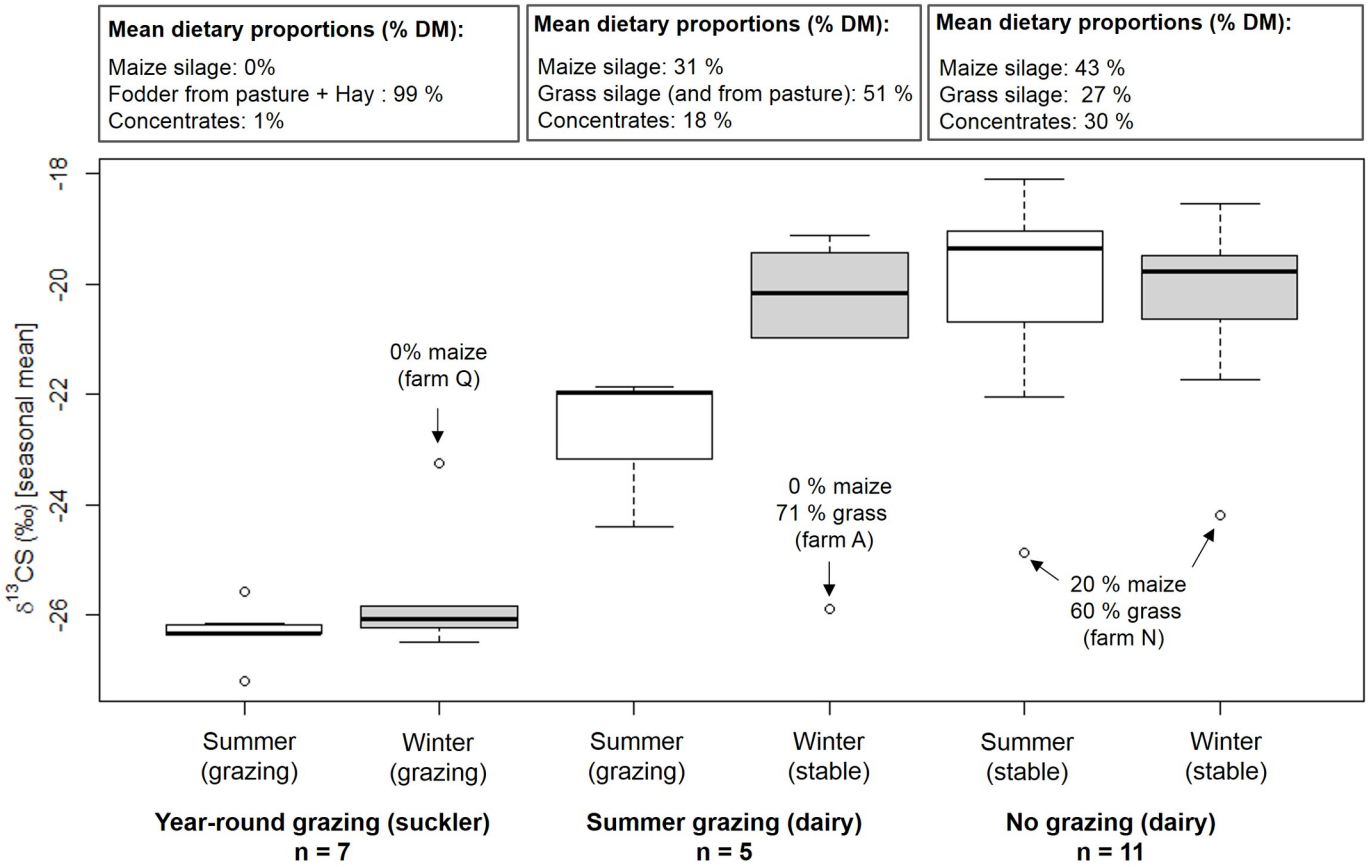
Box plots of δ^13^CS values of cattle hair sections analyzed for summer and winter of the three grazing regimes. The box represents the middle 50% of the data (between the 25th and 75th percentiles); the line in the box represents the median, the upper and lower lines represent the extreme values. Circles indicate outliers (>1.5 interquartile range) in the δ^13^CS value distributions within each season of each grazing regime. Annual dietary proportion (%) of maize, grass and concentrates are indicated in the three boxes above each grazing regime.

In order to show the sensitivity of the δ^13^C approach, we gave seasonal δ^13^CS in box plots (Fig 3) as these indicate variability within the groups as well as possible outliers. This approach enabled us to identify farms whose summer or winter δ^13^CS in cattle hair deviated strongly from the average seasonal δ^13^CS value of the respective grazing regime. The outliers for the no grazing and summer grazing regimes (farms A and N) could be explained by the annual dietary proportion of maize of the respective farms that deviated strongly from the annual dietary proportion of maize of the respective grazing regime (Fig 3).

The δ^13^CS value of -23.25 ‰ (winter period) in cattle hair of farm Q (year-round grazing regime) could also be explained by maize in the diet (Fig 3). In the interview at the time of sampling of the tail hairs, the farm manager of farm Q reported not to have fed maize in the previous year. However, after calling the farm manager again after data analysis and asking for a possible explanation of the high δ^13^CS in cattle hair, he remembered having fed maize silage from a neighboring farm for some time during November 2013.

The two outliers of the summer δ^13^CS in cattle hair in the year-round grazing regime were -27.18 ‰ and -25.57 ‰ and therefore below the -25.10 ‰- level that we discovered for the detection of maize in the cattle diet (see upper limit of CI of the linear model).

## Discussion

### Tracing back cattle diets using δ^13^C values in cattle hair

Our results confirm the close relationship between the dietary proportion of maize and δ^13^C in cattle hair that has previously been observed by other studies [21, 38, 44]. The critical value of an annual mean of -25.10 ‰ δ^13^C in cattle hair indicating maize in the cattle diet of up to one year before sampling of tail hair in our study falls well in the range of differences in δ^13^C in cattle hair of -25.00 ‰ to -27.00 ‰ that has previously been reported as possible values when cattle are fed with C_3_ plants from pastures [28]. Also concentrates solely derived from C_3_ plants have been found to result in similar or only slightly and insignificantly higher δ^13^C in cattle hair than C_3_ plants found in permanent pastures [6]. Furthermore, when considering trophic shifts from forage to cattle hair and from forage to milk casein [21, 45], the results of our model are in agreement with the range of values reported in studies on δ^13^C values of milk casein of cattle fed in a diet without maize (23.5–27.1 ‰) [18].

As expected, we found no significant difference in δ^13^CS values between the summer and winter season in the year-round grazing and no grazing regime. Cattle are normally not exposed to diet changes in these feeding regimes. However, also in the summer grazing regime where the cattle are supposed to ingest more C_3_ plants in summer than in winter due to the time they spent grazing on pastures, we found no significant difference in δ^13^CS values between seasons. This might be explained by the additional maize feeding during summer in this grazing regime as grazing is usually not full time (Table 1). Another point in this context concerns the sampling of cattle hair. Cattle tail hair of dairy cows was slightly too short to obtain the optimal hair section that would purely represent the isotopic signal from the summer period. As a consequence, isotopic information from the winter period was to a small extent present in the sections for summer. This needs to be considered when comparing the isotopic signals from summer and winter.

The two outliers of the summer δ^13^CS in cattle hair in the year-round grazing regime (Fig 3, -27.18 ‰ and -25.57 ‰) were below the -25.10 ‰- level and thus cannot be related to maize or other C_4_ plants in the diet [28]. The fact that we were able to detect distinct differences in δ^13^CS in cattle hair among the different grazing regimes and that even the temporary change in diet on farm Q could be revealed by analyzing the distribution of δ^13^CS values in cattle hair of distinct grazing regimes prove that even under practical conditions the approach of relating δ^13^C in cattle hair with annual dietary proportions of maize is a robust method for verifying feeding regimes, but also sensitive enough for detecting short-term deviations. Also for horses short-term diet changes could be detected in hair even if the isotopic signal is weaker compared to signals that result from a permanent change of diet [27].

The ability to partly reconstruct diets within a certain time span in the past using δ^13^C in cattle hair is a valuable tool to check, for example, for maize or other C_4_ plants in feeding regimes that are supposed to be completely based on grass or are grazing-only dairy systems [46]. Being able to estimate the amount of maize in the diet would be one step further than just distinguishing between grass-only systems and feeding systems that contain grass, maize and concentrates.

### Factors that can contribute to unexplained variance

In our study, 89% of the variance in the annual mean δ^13^C (δ^13^CY) in cattle hair could be explained by the dietary proportion of maize. However, there is a range of other factors which possibly could contribute to the unexplained 11%. Generally, plant communities that consist of C_3_ plants are sensitive to water availability and increasing precipitation results in reduced δ^13^C in cattle hair or hair of other grazers [28, 47–51]. Our study sites were chosen on a climatic gradient from sub-maritime to sub-continental. Differences in temperature and precipitation among our sites were in the range of Δ-temp.: 0.4°C and Δ-precip.: 168 mm (Table 2). For an investigation of the possible relationship between precipitation and δ^13^C in cattle hair we used data from farms which only fed C_3_ plants. We did not find any correlation between the annual precipitation or precipitation during the vegetation period and the δ^13^CY values in cattle hair, indicating an only weak relationship in our sample on farms along our climatic gradient. Studies that reported a significant influence of climatic parameters on δ^13^C values of plants of temperate grassland actually had more pronounced differences in temperature and precipitation among their sites with Δ-temp. of 0.9°C and Δ-precip. of 436 mm [28]. Soil texture directly determines the amount of water in the soil that is available to plants [52]. The effect of soil conditions on δ^13^C values of C_3_ plants is even stronger than that of climatic factors [28]. Therefore, although all farms were located on sandy soils, small differences in soil conditions like water retention could have contributed to the unexplained variance.

It is unlikely that the use of concentrates might have contributed decisively to the variation in δ^13^C in cattle hair. Studies on dairy farms that investigated the effect of concentrates from C_3_ plants on δ^13^C in cattle hair found very small differences in the isotopic signal [6, 21, 38, 53]. Moreover, concentrates derived from C_3_ plants and grass silage differ only very little in δ^13^C values (< 0.3 ‰) [6].

Some of the unexplained variance might also be related to inaccurate information on the composition of the cattle diet given by the farmer [36]. This is important as it served as the reference value for relating δ^13^C in cattle hair and maize in the diet. In our study we aimed at getting reliable information from the farmers by carefully preparing the interviews. We provided the relevant information of the survey to the farmers beforehand, we established a careful cross checking of data at all stages of data acquisition and analyses, and we contacted the farmers again in case of implausible data.

### Potential and limitations of using δ^13^C in cattle hair to trace back reported dietary proportions of maize in practice—What to keep in mind

We investigated differences between summer and winter δ^13^CS in cattle hair for 2013 by analyzing two centimeters of each anagen cattle hair sampled. The length of the tail hair, however, limits the time in the past from which the isotopic signal can be analyzed. To obtain clear isotopic information for the summer and winter periods of the same year, the tail hair needs to be at least 12 cm long and plucking time no later than March 15 of the following year. If the plucking time is much later than March 15, the previous summer would not be covered; if the plucking time is much earlier, the hair would only contain isotopic information of the summer period but not of the winter period of that year.

## Conclusion

In temperate areas, where pastures normally consists only of C_3_ plants, the δ^13^C signature in cattle tail switch hair is a reliable tool to be used in practice to retrospectively estimate the annual dietary proportion of maize in the previous year without analyzing the feed components. δ^13^C in cattle hair proved to be a robust indicator and the method can be applied in a range of cattle production systems. Sampling cattle tail hair of >11 cm length in spring can provide two different sections containing information on the feeding regime from the previous summer and winter periods. This information can be used to evaluate different grazing regimes (year-round grazing, summer grazing, no grazing) and also short term diet changes in the composition of feed (C_3_/C_4_ plants) in the cattle diet.

## Supporting information

S1 Dataset (XLSX)
